# Salinity Effects on Strategies of Glycogen Utilization in Livers of Euryhaline Milkfish (*Chanos chanos*) under Hypothermal Stress

**DOI:** 10.3389/fphys.2018.00081

**Published:** 2018-02-12

**Authors:** Chia-Hao Chang, Jian-Jun Huang, Chun-Yi Yeh, Cheng-Hao Tang, Lie-Yueh Hwang, Tsung-Han Lee

**Affiliations:** ^1^Department of Life Sciences, National Chung Hsing University, Taichung, Taiwan; ^2^Department of Oceanography, National Sun Yat-sen University, Kaohsiung, Taiwan; ^3^Mariculture Research Center, Fisheries Research Institute, Council of Agriculture, Yulin, Taiwan; ^4^Agricultural Biotechnology Center, National Chung Hsing University, Taichung, Taiwan

**Keywords:** glycogen phosphorylase, milkfish, liver, low temperature, seawater, fresh water

## Abstract

The fluctuation of temperature affects many physiological responses in ectothermic organisms, including feed intake, growth, reproduction, and behavior. Changes in environmental temperatures affect the acquisition of energy, whereas hepatic glycogen plays a central role in energy supply for the homeostasis of the entire body. Glycogen phosphorylase (GP), which catalyzes the rate-limiting step in glycogenolysis, is also an indicator of environmental stress. Here, we examined the effects of salinity on glycogen metabolism in milkfish livers under cold stress. A reduction of feed intake was observed in both freshwater (FW) and seawater (SW) milkfish under cold adaptation. At normal temperature (28°C), compared to the FW milkfish, the SW milkfish exhibited greater mRNA abundance of the liver isoform of GP (*Ccpygl*), higher GP activity, and less glycogen content in the livers. Upon hypothermal (18°C) stress, hepatic *Ccpygl* mRNA expression of FW milkfish surged at 3 h, declined at 6 and 12 h, increased again at 24 h, and increased significantly after 96 h. Increases in GP protein, GP activity, and the phosphorylation state and the breakdown of glycogen were also found in FW milkfish livers after 12 h of exposure at 18°C. Conversely, the *Ccpygl* transcript levels in SW milkfish were downregulated after 1 h of exposure at 18°C, whereas the protein abundance of GP, GP activity, and glycogen content were not significantly altered. Taken together, under 18°C cold stress, FW milkfish exhibited an acute response with the breakdown of hepatic glycogen for maintaining energy homeostasis of the entire body, whereas no change was observed in the hepatic glycogen content and GP activity of SW milkfish because of their greater tolerance to cold conditions.

## Introduction

Glycogen, a polymer of glucose residues, is a crucial form of energy storage. Catabolism of stored glycogen (i.e., glycogenolysis) occurs through the action of glycogen phosphorylase (GP; EC 2.4.1.1), which releases glucose-1-phosphate from the glycogen polymer and provides glucose for energy required by the organisms. GP is a homodimeric enzyme subjected to allosteric control and exhibits transitions between “relaxed” (active) and “tense” (inhibited) conformational states (Johnson, 1992; Agius, 2015). In fish, three isoforms of GP were named according to the tissues in which they occur, including the brain (*pygb*), liver (*pygl*), and muscle (*pygm*). The liver and skeletal muscle are two major tissues for glycogen storage. The concentration of glycogen is higher in the liver than in the muscle (10 vs. 2% by weight) (Berg et al., 2002). In the liver, glycogen degradation and synthesis play major roles in regulating blood glucose homeostasis and supplying energy to other tissues (Vornanen and Haverinen, 2011; Polakof et al., 2012).

Elevated blood glucose levels and energy metabolism have been detected in response to stress or environmental fluctuations (Lin et al., 2011; Polakof et al., 2012; Huang et al., 2015). Water temperature is critical to the physiological responses and energy utilization strategies of teleosts. Among different species, the best physiological performance (ex. behavior, feed intake, digestion, growth, and reproduction) was usually found in the optimal ranges (Pörtner et al., 2007; Handeland et al., 2008; Pörtner, 2009; Schram et al., 2013; Payne et al., 2016). Total GP activity of the liver and heart of the crucian carp (*Carassius carassius*) increased during the summer (Vornanen and Haverinen, 2011). In addition, a two-stage decline in temperature is the main trigger for active foraging and synthesis of glycogen before winter dormancy in the crucian carp, whereas the hepatic glycogen store did not change when the temperature declined steadily (Varis et al., 2016). The rainbow smelt (*Osmerus mordax*) is a temperate species and can tolerate low temperatures from 0.4 to −1.5°C. The production of glycerol is important as an antifreeze in the plasma and other tissues, and at 0.4°C, the increase in total GP activity promoted the breakdown of glycogen allowing glycerol accumulation in the rainbow smelt (Clow et al., 2008). Conversely, at 8°C, the gilthead sea bream (*Sparus aurata*) typically undergoes a multi-organ dysfunction called the “winter syndrome.” Furthermore, a reduction in feed intake and accumulation of liver glycogen were detected during pre-winter periods (18°C) (Couto et al., 2008; Ibarz et al., 2010c).

The milkfish (*Chanos chanos*) is an economically important species in Southeast Asia and Taiwan. The average temperatures in summer and winter in Taiwan are approximately 28°C and 18°C, respectively. Cold snaps in winter, however, sometimes make the temperature lower than 15°C, leading to high mortality of this tropical euryhaline species and causing huge economic losses. Acclimation of milkfish to seawater (SW) or fresh water (FW) induces a variety of physiological responses, including changes in their tolerance to hypothermal stress. SW milkfish exhibited better hypothermal tolerance than FW milkfish (Kang et al., 2015). The FW milkfish exhibited higher gill Na^+^/K^+^-ATPase activity that might require a greater energy supply compared to that of the SW individuals (Lin et al., 2003; Kang et al., 2015). From transcriptome analyses of hypothermal milkfish (Hu et al., 2015, 2017), the fragments per kilobase of transcript per million reads mapped (FPKM) of GP was found to increase 1.28-fold in FW individuals and decrease to 0.77-fold in SW fish. Moreover, the protein abundance of lactate dehydrogenase was found to be upregulated in the liver of SW milkfish under hypothermal adaptation, indicating the requirement of a larger energy source under the stressful conditions (Chang et al., 2016b). Hence, the strategies of energy utilization in livers between FW and SW milkfish are proposed to be distinct, leading to different cold tolerance upon hypothermal stress.

Being an important factor for energy supply of fish, the feed intake was found to be lower in the gilthead sea bream under cold stress (Ibarz et al., 2007; Couto et al., 2008). The decreased energy source from feed intake led to a degradation in hepatic glycogen for maintaining blood glucose (Ibarz et al., 2010a). In this study, feed intake and hepatic glycogen content were analyzed in FW and SW milkfish under hypothermal (18°C) stress. In addition, the partial sequence of *Ccpygl* was identified. The *Ccpygl* mRNA abundance and GP activity in the livers of 18°C-exposed FW and SW milkfish were compared. Our data illustrated distinct strategies of glycogen utilization corresponding to different low temperature tolerances for FW- and SW-acclimated euryhaline milkfish.

## Materials and methods

### Rearing conditions of milkfish

Juvenile milkfish were obtained from a local fish farm in Lukang, Taiwan. Fish were transported to the laboratory, where they were raised in four 400 L rearing tanks; two with seawater (SW 35‰) and two with FW, at 28 ± 1°C with a daily 12 h photoperiod for at least 1 month to reach a steady state (Kang et al., 2013; Chang et al., 2016c; Hu et al., 2017). The water for rearing tanks was continuously circulated through fabric-floss filters, and salinity was measured by a refractometer ATC-S (ATAGO, Tokyo, Japan). All experimental animals were fed to satiation at 15:00–16:00 everyday. The milkfish commercial diets contain 24% crude protein and 3% crude lipid (FWUSOW Industry, Taichung, Taiwan). In total, 188 milkfish were used in the present study. The protocol for the experimental fish was reviewed and approved by the Institutional Animal Care and Use Committee (IACUC) of the National Chung Hsing University (IACUC Approval No. 105-024 to THL).

### Hypothermal acclimation/stress experiments

For these two experiments, the temperature was maintained at 28 ± 1°C for the control group and 18 ± 0.5°C for the hypothermal group. The water for hypothermal SW and FW groups was cooled down at a constant rate (2°C h^−1^) with a cooling system (PF-225M, PRINCE, Tainan, Taiwan). After transfer to the 100 L experimental tanks with a hypothermal (18°C) or control temperature (28°C) from the 400 L rearing tanks, the milkfish were maintained and stabilized in the experimental tanks for at least 2 days. For the hypothermal acclimation experiments, milkfish were kept in four 100 L experimental tanks/conditions with different temperature × salinity set-ups ([1] SW/28°C, [2] FW/28°C, [3] SW/18°C, and [4] FW/18°C) for 1 week and then sampled (*n* = 6 for each condition). For the hypothermal stress experiments, milkfish were acclimated to 28°C followed by a temperature drop to 18°C at a rate of 2°C per hour, and subsequent sampling at 1, 3, 6, 12, 24, 48, 96, and 168 h after reaching 18°C (*n* = 6 for each of the salinities). During the experiments, milkfish were fed once per day. The experimental fish were euthanized the morning after the end of the experiment and anesthetized with 0.5% 2-phenoxyethanol before sampling. All efforts were made to minimize suffering and distress. The average body weight and average total length of sampled milkfish were 13.1 ± 2.3 g and 10.2 ± 1.3 cm, respectively. Milkfish livers were dissected quickly, immersed in liquid nitrogen, and stored at −80°C until the following analyses.

### Feed-intake experiments

In addition to the hypothermal acclimation/stress experiments, 32 experimental milkfish were used in feed-intake experiments. Four experimental conditions ([1] SW/28°C, [2] FW/28°C, [3] SW/18°C, and [4] FW/18°C) of the feed-intake experiments were performed, and eight experimental animals were used in each condition. For each experimental condition, a single milkfish was reared in a 100 L experimental tank for 1 week and used for feed-intake measures only once. During the experimental period, this milkfish in the experimental tank was fed once (15:00–16:00) daily. On the 17 day of the experiment, the feed-intake of the milkfish in the experimental condition was evaluated. Several 1.5 mL-tubes of feed pellets were prepared before feeding. 0.02 g milkfish commercial feed pellets that were stable in water were first weighed and packed in each 1.5 mL tube, and the number of feed pellets in the tube was counted. After feeding, all amounts (tubes) of feed pellets and the pellet residue in the experimental tank were counted. The uptake of feed pellets of each experimental milkfish per day (g fish^−1^ day^−1^) was worked out by subtracting the amount of pellet residue in the tank from the number of feeding pellets and converting to weight (g) of feed. The average feed-intake of milkfish in each condition was derived from eight individuals.

### Total RNA extraction and reverse transcription

Total RNA samples were isolated using the Tripure Isolation Reagent following the manufacturer's instructions. The genomic DNA contamination in RNA samples was eliminated by using the RNAspin Mini RNA isolation kit (GE Healthcare, Piscataway, NJ, USA). RNA integrity was verified by electrophoresis in 1% agarose-gel. The purity and concentration of extracted RNA were measured with a NanoDrop 2000 (Thermo Fisher Scientific, Waltham, MA, USA). Purified RNA with an A260/A280 ratio of between 1.8 and 2.0 was used for the following experiments. First-strand cDNA was synthesized by reverse transcribing 1 μg of the total RNA and the iScript Reverse Transcription Supermix (Bio-Rad Laboratories, Hercules, CA, USA) was used according to the manufacturer's instructions. The cDNA samples were stored at −20°C before analyses.

### cDNA cloning and sequence analysis

The partial sequence of *Ccpygl* was identified (KY923199), and the primers were designed by the Primer 3 Plus based on highly conserved regions compared with other teleosts from the NCBI database (Table 1). For PCR amplification, 2 μL of cDNA from the milkfish liver was used as the template in a 50 μL PCR reaction containing 0.25 μM dNTPs, 2 U of Ex-Taq polymerase, and 0.1 μL of cloning primer. PCR products were ligated into the pGM-T vector and sequenced. The amino acid sequence of GPL was used to build a phylogenetic tree using MEGA 6, and the tree was built using the maximum likelihood method in 1,000 bootstraps.

**Table 1:** Reference proteins from NCBI database for Pygl phylogenetic analysis.

**Species**	**Gene name**	**Accession number**
*Astyanax mexicanus*	PREDICTED: glycogen phosphorylase liver form	XP 006632545.1
*Cynoglossus semilaevis*	PREDICTED: glycogen phosphorylase liver form	XP 008311194.1
*Danio rerio*	Glycogen phosphorylase, liver form	NP001008538.1
*Fundulus heteroclitu*	PREDICTED: glycogen phosphorylase liver form	XP 012727698.1
*Haplochromis burtoni*	PREDICTED: glycogen phosphorylase liver form	XP 005946111.1
*Homo sapiens*	Liver glycogen phosphorylase Glycogen phosphorylase, liver form isoform 1 Liver glycogen phosphorylase	AAC17450.1 NP002854.3 AAC23504.1
*Ictalurus punctatus*	Glycogen phosphorylase, liver form	AHH39573.1
*Larimichthys crocea*	Glycogen phosphorylase, liver form PREDICTED: glycogen phosphorylase liver form	KKF13421.1 XP 010752900.1
*Lepisosteus oculatus*	PREDICTED: glycogen phosphorylase liver form	XP 006632545.1
*Mus musculus*	Glycogen phosphorylase, liver form Liver glycogen phosphorylase	NP573461.2 AAH13636.1
*Maylandia zebra*	PREDICTED: glycogen phosphorylase liver form	XP 004540100.1
*Neolamprologus brichardi*	PREDICTED: glycogen phosphorylase liver form	XP 006798715.1
*Oryzias latipes*	PREDICTED: glycogen phosphorylase liver form	XP 004082115.1
*Oreochromis niloticus*	PREDICTED: glycogen phosphorylase liver form	XP 003442910.1
*Poecilia Formosa*	PREDICTED: glycogen phosphorylase liver form	XP 014849665.1
*Poecilia latipinna*	PREDICTED: glycogen phosphorylase liver form	XP 014887185.1
*Pundamilia nyererei*	PREDICTED: glycogen phosphorylase liver form	XP 005736772.1
*Rattus norvegicus*	Glycogen phosphorylase, liver form	NP071604.1
*Salmo salar*	Glycogen phosphorylase, liver form	XP014067148.1
*Scleropages formosus*	Glycogen phosphorylase, liver form	KPP79726.1
*Stegastes partitus*	PREDICTED: glycogen phosphorylase liver form	XP 008298301.1
*Takifugu rubripes*	PREDICTED: glycogen phosphorylase liver form	XP 003962378.1

### Real-time PCR

The mRNA expression was detected by KAPA SYBR FAST qPCR Kit Master Mix and quantified with the Mini Opticon real-time PCR system. The amplification efficiencies of the primers (Table 2) were evaluated to be 90–105%, and the *r*^2^ of the serial dilutions was evaluated to be 0.99. A single peak appeared in the melting curve analyses and the presence of a single amplification product was observed using 1.5% agarose gel. The mRNA expression of *Ccgpl* were normalized with the *Chanos chanos* 60S acidic ribosomal protein P2 (*Ccrplp2*) gene from the same cDNA sample. The expression levels of *Ccrplp2* were not significantly different among various groups (Table 3). The PCR reactions contained 8 μL of cDNA, 2 μL of qPCR primer (2 μM), and 10 μL of SYBR Master Mix. The liver samples of the four experimental groups (FW/28°C, FW/18°C, SW/28°C, and SW/18°C) were pooled and used as the internal control (IC) among different qPCR analyses. The relative gene expression formula was 2^∧^–[(Ct_*Ccgpl*, n_ – Ct_*Ccrplp*2, n_) – (Ct_*Ccgpl*, IC_ – Ct_*Ccrplp*2, IC_)] (Livak and Schmittgen, 2001).

**Table 2:** Primer sequences used in this study.

**Gene**	**Primers**	**Sequence (5′-3′)**	**Application**
*Ccpygl*	F793	AAC ACAATG AGG CTG TGG TC	PCR
	R2458	GAG TGG ACG AAG ATG GTG AT	
	qF24	GAC TTT AAC GTC GGC GAT TAC	qPCR
	qR158	GAA GCA GGA GTA TTT TGT GGT G	
*Ccrplp2*	qF	GTT ACC GGT CGG CCG TTG A	qPCR
	qR	AGA AGT CCA AAA AAG GAG CTT CCT	

**Table 3:** The Δ*Ct* value of mRNA expression of *Ccrplp2* in liver milkfish.

	**FW/28°C**	**FW/18°C**	**SW/28°C**	**SW/18°C**
*Ccrplp2*	1.40 ± 0.25	1.16 ± 0.22	1.36 ± 0.19	1.14 ± 0.14

*Values are means ± SEM, n = 6. There was no significant difference among various groups*.

### Immunoblotting

The immunoblotting protocol was modified from Chang et al. (2016c). The milkfish livers were suspended in SEID medium (150 mM sucrose, 10 mM EDTA, 50 mM imidazole, 0.1% sodium deoxycholate; pH 7.5) containing protease inhibitor (vol/vol: 25:1; Roche, Mannheim, Germany) and were homogenized with a Polytron PT1200E homogenizer (Lucerne, Switzerland) at maximum speed. The homogenates were then centrifuged at 10,000 × g at 4°C for 10 min. Protein concentrations of the supernatants were determined using reagents from the Protein Assay Kit (Bio-Rad), and bovine serum albumin (Sigma-Aldrich, St. Louis, MO, USA) was used as a standard. The homogenates containing 50 μg were heated at 60°C for 15 min and fractionated by electrophoresis on SDS containing 8% polyacrylamide gels. The pre-stained protein molecular weight marker (#26616, Thermo) was applied in electrophoresis. The separated proteins were transferred to 0.45 μm PVDF blotting membranes (Millipore, Bedford, MA, USA). The PVDF membranes were incubated for 1 h in PBST with 5% (wt/vol) nonfat dried milk to minimize non-specific binding. The blots were incubated with the primary antibody (GP, 1:10,000; GTX124390; Genetex, Irvine, CA, USA; GAPDH, 1:5,000; GTX100118; Genetex) overnight at 4°C, followed by incubation with the HRP-conjugated secondary antibody (goat anti-rabbit IgG, 1:10,000; GTX213110; Genetex) for 1 h at 28°C. The blots were developed with the Immobilon Western Chemiluminescent HRP substrate (Millipore). The images were photographed using the universal hood with a cooling-charge-coupled device (CCD) camera (ChemiDoc XRS^+^, Bio-Rad) and analyzed with ImageLab software version 3.0 (Bio-Rad) to normalize numerical values compared to the relative intensities of immunoreactive bands. The protein of GP was detected as a single immunoreactive band at 93 kDa (Figure S1).

### Glycogen phosphorylase (GP) activity

Functional GP is capable of binding to glycogen when the enzyme is in the R (relaxed) state. The phosphorylated GP is automatically changed to the R state, whereas unphosphorylated GP in the T (tense) state is regulated by AMP of the allosteric effector and then transformed to the R state. The total GP activity consists of detected activity of both the R (determined by AMP^−^ assay buffer) and T (determined by AMP^+^ assay buffer) states (Johnson, 1992; Clow et al., 2008; Vornanen and Haverinen, 2011; Bolinger and Rodnick, 2014; Agius, 2015). The GP activity was determined according to the NAPDH-linked method with modification. The direction of glycogenolysis by determination of NAPDH was enzymatically coupled to phosphoglucomutase and glucose-6-phosphate dehydrogenase. The liver from each milkfish was dissected quickly and immersed in liquid nitrogen. A 10 mg sample of liver tissue was rapidly homogenized in 1 mL buffer (100 mM imidazole, 100 mM NaF, 2 mM EDTA, 0.1 mM PMSF) using a Polytron PT1200E at the maximal speed for 5 s on ice. The homogenates were centrifuged at 5,000 × g, at 4°C for 5 min. The supernatants were used for the GP activity assay. The GP activity assay solution (50 mM potassium-phosphate buffer, 0.2 mg/mL glycogen, 0.1 mM EDTA, 15 mM MgCl_2_, 6.5 mM NADP^+^, 0.001 mg/mL glucose-1,6-bisphosphate, 10 U/mL phosphoglucomutase, 10 U/mL glucose-6-phosphate dehydrogenase) was the 5′AMP^−^ assay buffer for determining GP activity in the R-state. Conversely, the 5′AMP^+^ assay buffer for determining total GP activity (R+T-state) was the GP activity assay solution plus 1.6 mM 5′AMP. The sample of 25 μL from each fish was loaded into each well, together with 175 μL of the 5′AMP^−^ or 5′AMP^+^ assay buffer. Each sample was assayed in triplicate. The 96-well microplate was analyzed every 1 min for up to 5 min in the VERSAmax microplate reader at 340 nm and 28°C. Serial dilution of NADPH (50, 25, 12.5, 6.25, and 3.125 μg in 25 μL sample buffer) were prepared for the standard curve.

### Glycogen contents

For quantification of glycogen, 50 mg of milkfish livers were homogenized in ddH_2_O. Then the homogenates were boiled for 10 min to inactive enzymes. The boiled samples were centrifuged at 18,000 × g for 10 min to remove insoluble material. The glycogen contents were subsequently determined using the glycogen colorimetric/fluorometric assay kit (K646-100, Biovision, Milpitas, CA, USA) following the manufacturer's instructions. Then, 5 μL of the 40 × dilution of liver samples were added to each well of the 96-well microplate, and the volume was brought to 50 μL with the hydrolysis buffer incubated at 28°C for 30 min. Then, development buffer mixture was added and incubated for 30 min at 28°C. Serial dilution of glycogen (0.04, 0.08, 0.12, 0.16, and 0.2 μg in 50 μL hydrolysis buffer) was prepared for the standard curve. The absorbance was measured in the VERSAmax microplate reader at 570 nm and the glycogen standard curve was used to calculate the glycogen concentration of the samples.

### Statistical analysis

Values are expressed as the mean ± SEM (standard error of mean). The quantitative values of 1-week data from hypothermal and control groups were determined by two-way analysis of variance (ANOVA) followed by Tukey's HSD *post-hoc* test on R version 3.4.2 (R foundation, Vienna, Austria). Data from time-course experiments were compared using the one-way ANOVA analysis with Dunnett's pairwise method on R version 3.4.2, using the 0 h data as the control. The significance level was set at *P* < 0.05.

## Results

### Hypothermal effects on feed intake between FW and SW milkfish

**Figure 1:**
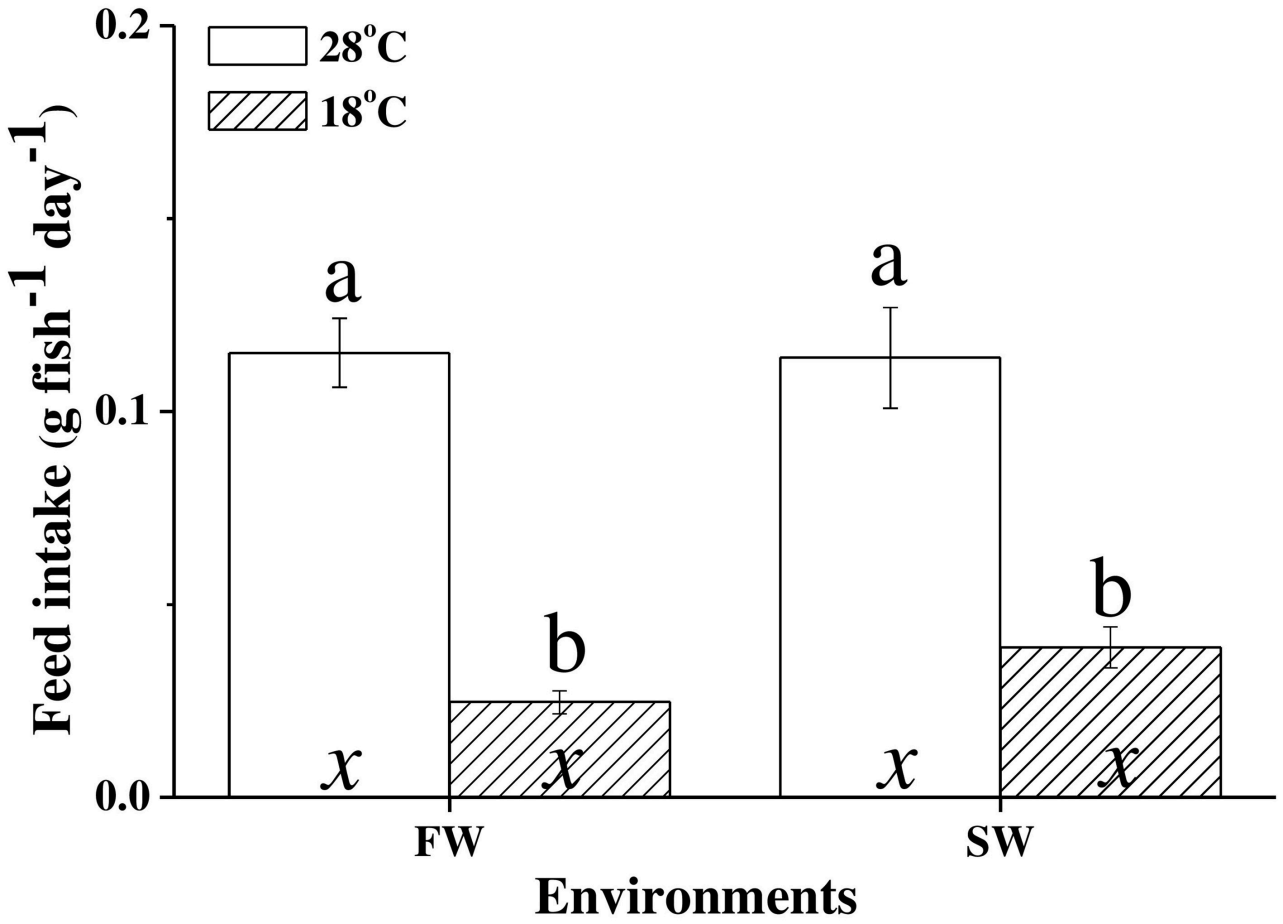
Feed intake (g fish^−1^ day^−1^) between hypothermal freshwater (FW) and seawater (SW) milkfish. Value are means ± SEM, *n* = 8. Different letters (a vs. b) indicate significant differences between the 28°C and 18°C group at the same salinity, and the letter “x” indicate no significant difference between the FW and SW group at the same temperature. The TukeyHSD pairwise comparison was used following two-way ANOVA, *P* < 0.05.

The feed intake of milkfish was significantly affected by water temperature; the average feed intake per day for FW- and SW-acclimated milkfish was 0.115 ± 0.009 g and 0.115 ± 0.013 g at 28°C, whereas it was reduced to 0.025 ± 0.003 g and 0.039 ± 0.005 g, respectively, under hypothermal acclimation (Figure 1). The two-way ANOVA analyses revealed that feed intake was affected by hypothermal acclimation, whereas the synergistic interaction did not significantly affect feed intake (Table 4).

**Table 4:** Results of two-way ANOVA evaluating the source of temperature and salinity variations in livers of milkfish.

	**Temperature**			**Salinity**			**Temperature** × **Salinity**		
	***F***	***df***	***P***	***F***	***df***	***P***	***F***	***df***	***P***
Feed intake	1.49	1, 28	<0.01^**^	60.09	1, 28	0.24	0.87	1, 28	0.35
*Ccpygl* expression	45.08	1, 20	<0.01^**^	34.88	1, 20	<0.01^**^	78.74	1, 20	<0.01^**^
GP protein abundance	1.815	1, 20	0.19	0.1359	1, 20	0.71	1.16	1, 20	0.30
GP activity	32.62	1, 20	<0.01^**^	0.6484	1, 20	0.43	27.36	1, 20	<0.01^**^
Glycogen content	118.16	1, 20	<0.01^**^	25.60	1, 20	<0.01^**^	75.31	1, 20	<0.01^**^

Df, degree of freedom; F, F statistic; ^*^ P ≤ 0.05;

** *P ≤ 0.01. Values in bold indicate significant differences*.

### Phylogenetic tree and tissue distribution of *Ccpygl*

**Figure 2:**
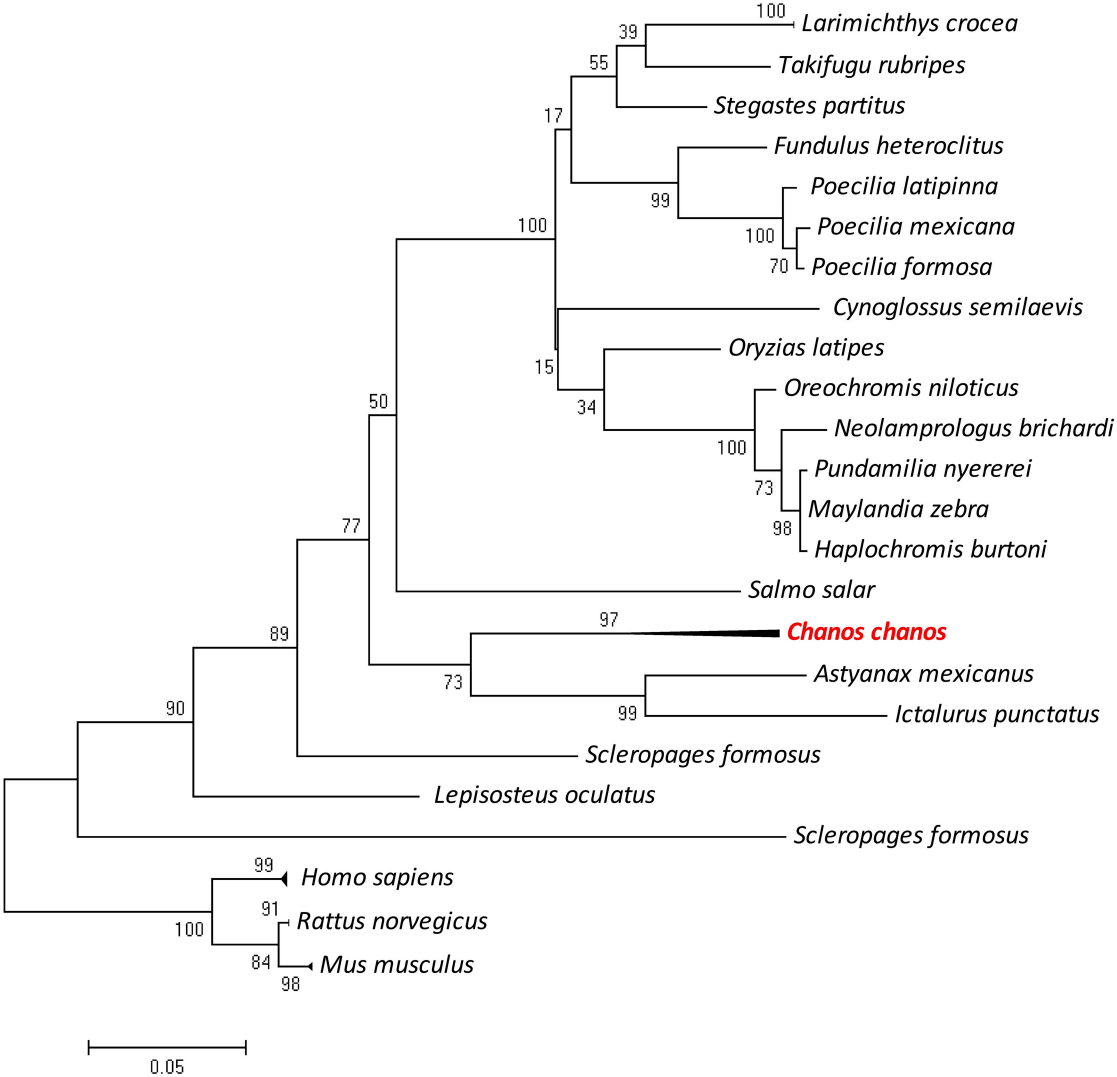
Phylogenetic analysis of milkfish (*Chanos chanos*) liver isoform of glycogen phosphorylase (Pygl) proteins based on amino acid sequences using the maximum likelihood method. The results were confirmed by 1000 bootstraps. Sequence accession numbers of different proteins are listed in Table 2.

**Figure 3:**
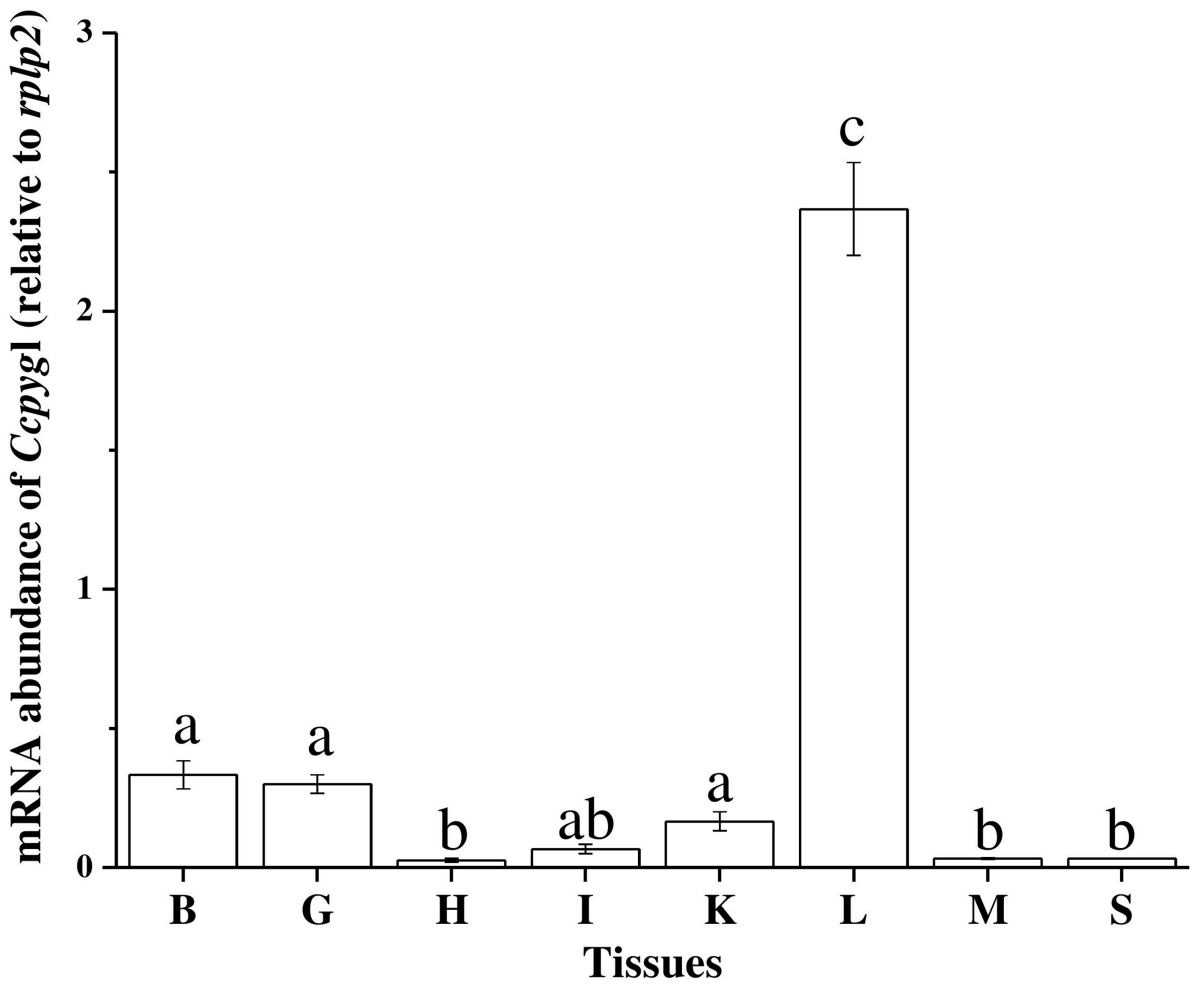
Tissue distribution of *Ccpygl* (*Chanos chanos* liver isoform of glycogen phosphorylase) mRNA expression of the milkfish detected by qPCR. Values are means ± SEM, *n* = 3. B, brain; G, gill; H, heart; I, intestine; K, kidney; L, liver; M, muscle; S, spleen.

The partial sequence of *Ccpygl* (1,606 bp, accession number: KY923199) was cloned from the liver of milkfish. This partial amino acid sequence was simultaneously confirmed to be the liver isoform of GP (*pygl*) by phylogenetic analyses in comparison with all three isoforms of GP (i.e., liver isoform, [*pygl*]; muscle isoform [*pygm*]; and brain isoform [*pygb*]) of other species (Figure S2). Subsequent phylogenetic analyses revealed that the amino acid sequence of Pygl of milkfish was highly similar to Pygl sequences of Mexican tetra (*Astyanax mexicanus*; 89%) and channel catfish (*Ictalurus punctatus*; 87%) and was categorized in the branch of *Ostariophysi* (Figure 2). Among the tissues collected from the SW milkfish, *Ccpygl* expression determined by qPCR was found to be primarily abundant in the liver. In addition to the liver, lower expression of *Ccpygl* was detected in the brain, gills, and kidney (Figure 3).

### Salinity effects on glycogen catabolism in the hypothermal acclimation experiments

**Figure 4:**
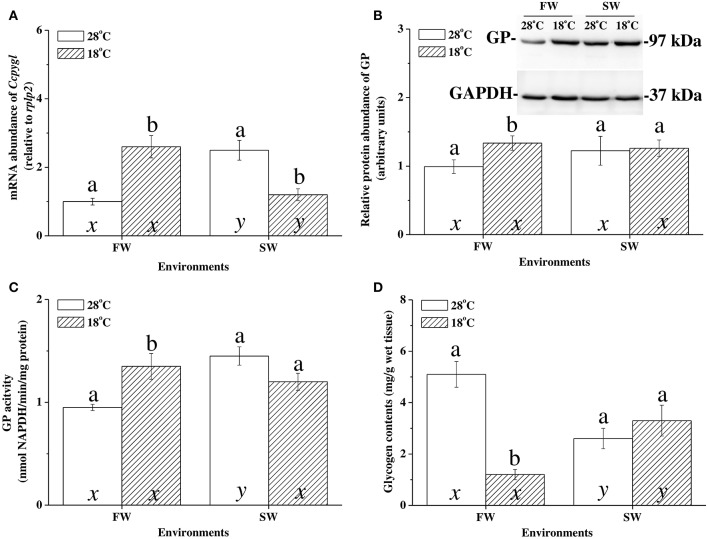
The hypothermal acclimation experiments revealed the glycogen catabolism pattern in livers of freshwater (FW) and seawater (SW) milkfish after 1-week hypothermal stress. **(A)** mRNA expression of *Ccpygl*, **(B)** abundance of GP protein, **(C)** GP activity, and **(D)** glycogen content. Values are means ± SEM, *n* = 6. Different letters (a vs. b and x vs. y) indicate significant differences between the 28°C and 18°C group at the same salinity, and the FW and SW group at the same temperature, respectively. The TukeyHSD pairwise comparison followed the two-way ANOVA, *P* < 0.05.

At 28°C, the mRNA expression of *Ccpygl* and GP activity were significantly higher in the livers of SW milkfish than in those of FW milkfish (Figures 4A,C). The glycogen storage in the livers of SW milkfish, however, was lower than in those of the FW group (Figure 4D). Conversely, in the hypothermal acclimation experiments, the mRNA expression profiles of *Ccpygl* in the livers were upregulated in FW milkfish and downregulated in SW fish (Figure 4A). Hepatic GP protein (Figure 4B) and GP activity (Figure 4C) were also increased in FW milkfish under hypothermal acclimation, but not significantly different than in hypothermal SW-acclimated individuals (Figures 4B,C). Meanwhile, in the hypothermal acclimation experiments, glycogen contents were found to downregulate in the livers of FW milkfish, whereas they were slightly, but not significantly (*p* = 0.247), elevated in the livers of SW milkfish (Figure 4D). The two-way ANOVA analyses revealed that *Ccpygl*, GP activity, and glycogen content were affected by synergistic interaction between temperature and salinity (Table 4).

### Acute changes in *Ccpygl* mRNA expression in the hypothermal stress experiments

**Figure 5:**
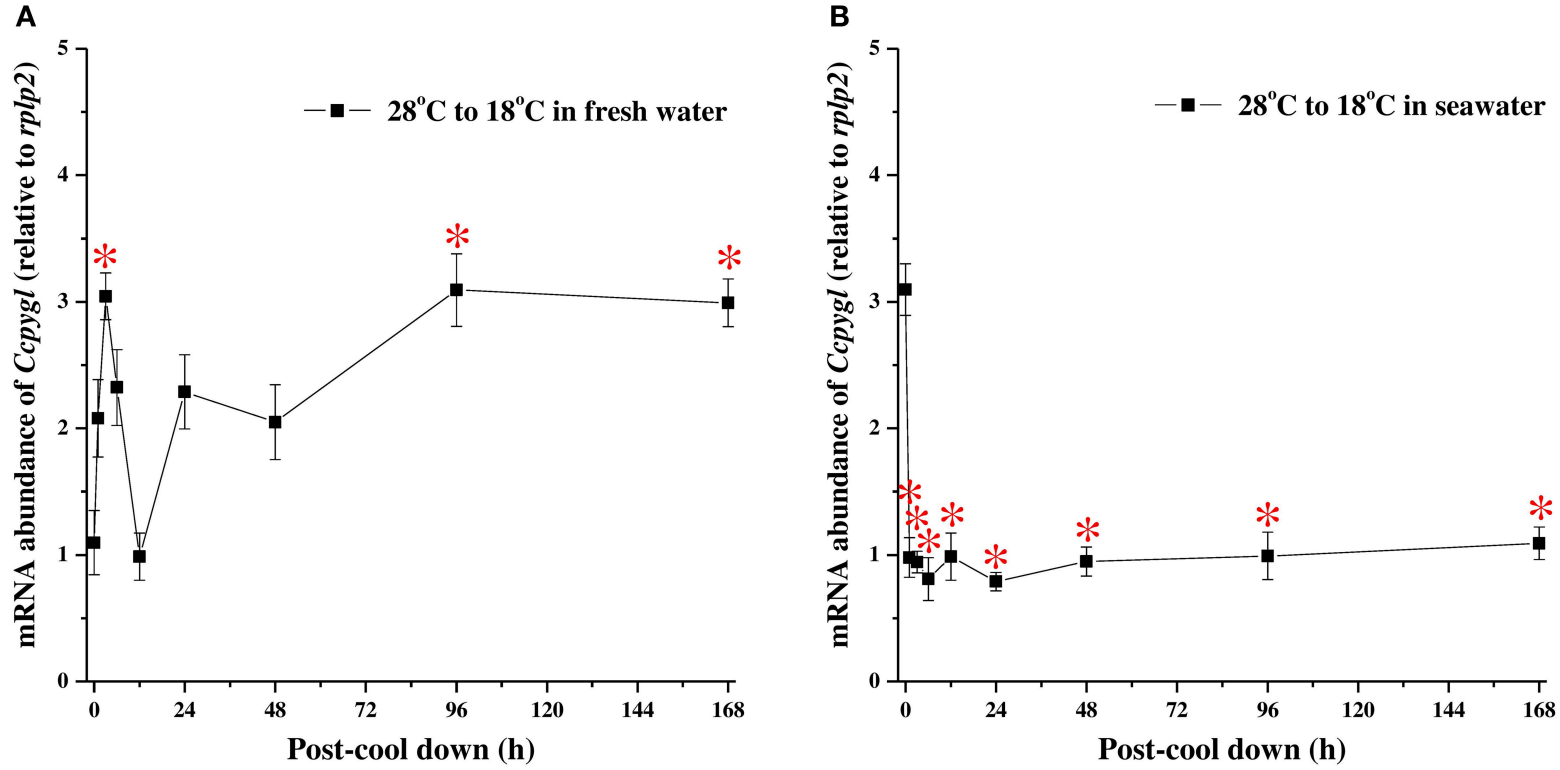
The hypothermal stress experiments revealed time-course mRNA expression of *Ccpygl* in livers of **(A)** fresh water (FW) and **(B)** seawater (SW) milkfish. Values are means ± SEM, *n* = 6. The asterisks indicate significant differences (*P* < 0.05) compared to the 0 h fish using one-way ANOVA with Dunnett's test.

The acute mRNA expression of *Ccpygl* in milkfish livers was determined by qPCR at 0 h (control) and after hypothermal stress for 1, 3, 6, 12, 24, 48, 96, and 168 h in FW (Figure 5A) or SW (Figure 5B). In hypothermal FW, the mRNA expression of *Ccpygl* responded in a biphasic manner with a rapid increase peaking at 3 h and a second elevation at 96 h post transfer (Figure 5A). Conversely, in hypothermal SW, a significant downregulation of *Ccpygl* mRNA abundance occurred from 1 h post transfer to the end of the hypothermal stress experiment (168 h post transfer) (Figure 5B).

### Different patterns of hepatic glycogen catabolism between FW and SW milkfish in the hypothermal stress experiments

**Figure 6:**
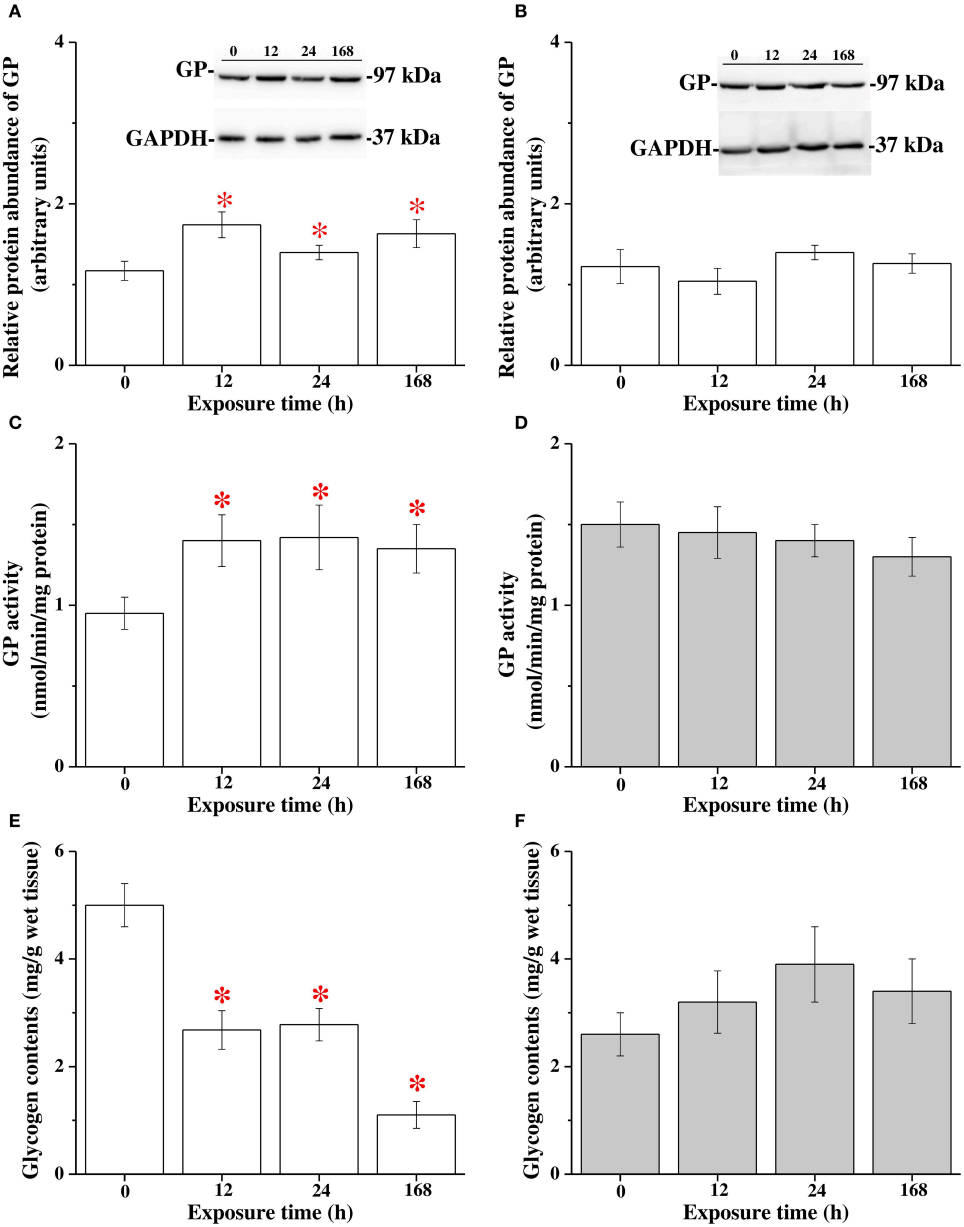
The hypothermal stress experiments revealed glycogen catabolism in livers of freshwater (FW) and seawater (SW) milkfish after 18°C-exposure. The protein abundance of GP in FW **(A)** and SW **(B)** groups, GP activity in FW **(C)** and SW **(D)** groups, glycogen contents in FW **(E)** and SW **(F)** groups, respectively. Values are means ± SEM, *n* = 6. The asterisks indicate significant differences (*P* < 0.05) compared to the 0 h fish using one-way ANOVA with Dunnett's test.

The protein abundance of GP, GP activity and glycogen contents in the livers of milkfish were analyzed at 0 (control), 12, 24, and 168 h of 18°C-exposure. The protein abundance of GP and GP activity in the livers of FW milkfish was significantly upregulated after 12, 24, and 168 h (approximately 1.5-fold) of 18°C-exposure (Figures 6A,C). In SW milkfish, however, no significant difference in protein abundance of GP and GP activity were found among different time-point groups in the hypothermal stress experiments (Figures 6B,D). Moreover, hepatic glycogen contents were significantly downregulated in FW milkfish after 12 h of hypothermal stress, and onward (Figure 6E). Conversely, in SW there was no significant change among different time-points after exposure to 18°C (Figure 6F).

### The percentage of the active form of GP in the hypothermal stress experiments

**Figure 7:**
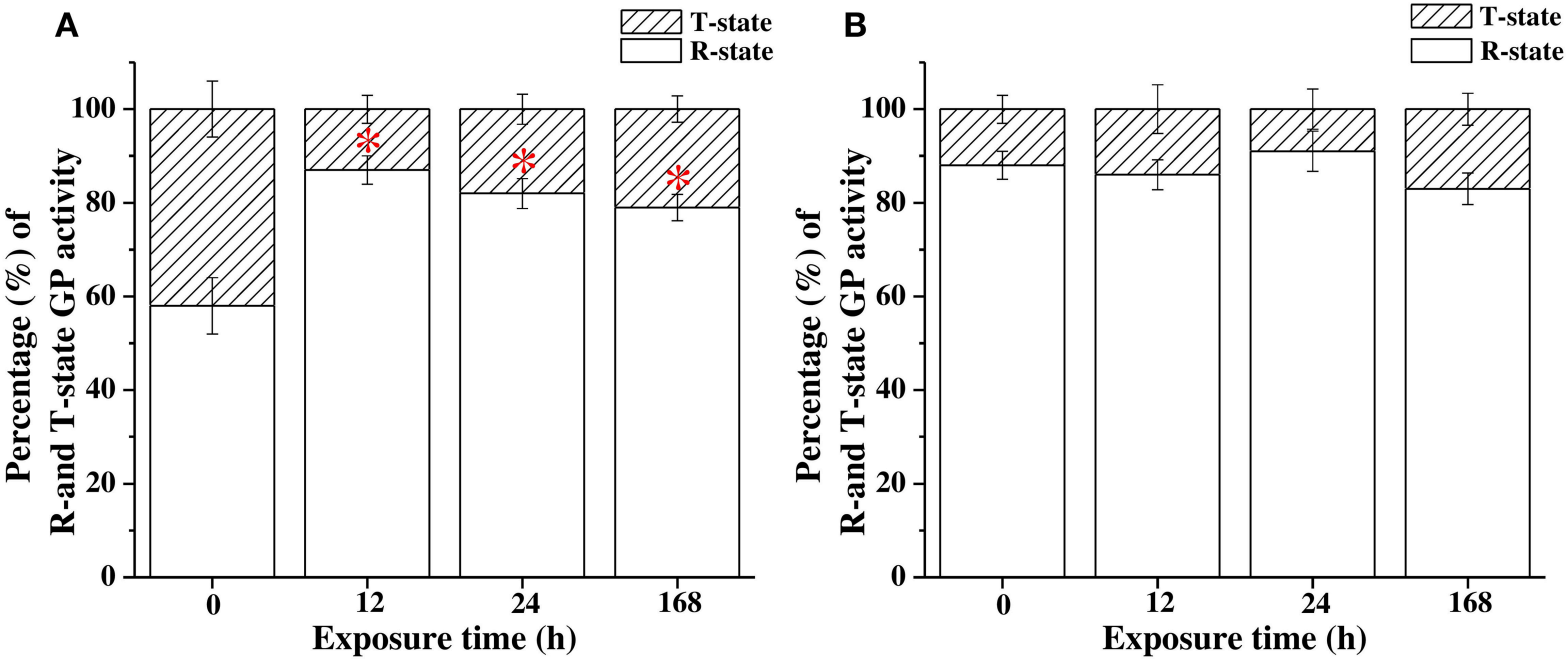
The percentages of active form of GP activity in livers of **(A)** freshwater (FW) and **(B)** seawater (SW) milkfish in the hypothermal stress experiments after 18°C-exposure. Values are means ± SEM, *n* = 6. The percentage of the activity of GP active form: GP activity (AMP^−^)/total GP activity (AMP^+^) × 100 (%). The asterisk indicates a significant difference compared to the 0 h fish by one-way ANOVA (Dunnett's comparison, *P* < 0.05).

The R-state is the phosphorylated state of GP with higher activity to degrade glycogen, whereas the T-state is the dephosphorylated state of GP with lower activity to breakdown glycogen. The percentage of the active form of GP activity was calculated as the results of GP (R-state; determined by AMP^−^ assay buffer) divided by the total GP activity (R + T-state; determined by AMP^+^ assay buffer). Increasing percentages indicated that the levels of phosphorylation of GP in FW milkfish were significantly upregulated after 12, 24, and 168 h of exposure to 18°C (Figure 7A). However, the phosphorylation level of GP was not significantly different to that in SW milkfish in the hypothermal stress experiments (Figure 7B).

## Discussion

The most important finding of the present study was that under hypothermal stress, salinity affected the glycogen utilization strategy of milkfish. The mRNA expression, protein abundance, and GP activity were upregulated and correlated with glycogen catabolized (correlation coefficient: mRNA, −0.88; protein, −0.60; GP activity, −0.81) in FW-acclimated milkfish at 18°C. Moreover, mRNA expression of *Ccgpl* behaved in a biphasic manner, with an early and late phase, and the protein abundance of GP and phosphorylation state of GP were regulated acutely in FW-acclimated individuals under hypothermal stress. The GP activity and glycogen contents, however, were not significantly altered in SW-acclimated milkfish when exposed to 18°C. Only the mRNA expression of *Ccgpl* was downregulated after 1 h exposure at 18°C.

### Phylogenetic relationship of glycogen phosphorylase in milkfish

Near et al. (2014) described the phylogenetic relationship of gonorynchiform fishes by using several nuclear DNA genes. The milkfish (*Chanos chanos*) is a primitive species originating in the Mesozoic and classified in Chanidae of Gonorynchiformes. The phylogenetic tree of ossification showed that milkfish was classified with the original ray-finned fish (Arratia and Bagarinao, 2010). The *pygl* gene of milkfish was classified in the Ostariophysi family. This gonorynchiform species is close to Characiformes (*Astyanax mexicanus*) and Siluriformes (*Ictalurus punctatus*) species (Arratia and Bagarinao, 2010). Three isoforms of GP were highly similar in their amino acid sequences. They were encoded by three genes, *pygl, pygm*, and *pygb*, which were mainly distributed in the liver, muscle, and brain, respectively (Tseng et al., 2007; Polakof et al., 2012). In the present study, the CcPygl sequence was confirmed according to the phylogenetic tree of these three isoforms.

### Salinity effects on glycogen utilization in the livers of milkfish

The liver is an important organ for energy metabolism, and is responsible for glycogen and glucose turnover, fatty acid synthesis, and gluconeogenesis in all teleosts (Polakof et al., 2012). Differentially expressed genes related to energy metabolism were recently reported in the livers of the gilthead sea bream (*S. aurata*), after hypo-osmotic challenge (Martos-Sitcha et al., 2016). The glycogen levels and GP activity were not changed under hypo-osmotic stress in the livers of the gilthead sea bream (Laiz-Carrión et al., 2005) as well as another marine species, the Senegalese sole (*Solea senegalensis*) (Arjona et al., 2009). Similarly, in freshwater goldfish (*Carassius auratus*), the levels of hepatic glycogen did not change under hyperosmotic stress (Luz et al., 2008). The hepatic glycogen contents of tilapia (*Oreochromis mossambicus*) and rainbow trout (*Oncorhynchus mykiss*) were utilized in an acute phase upon hyperosmotic challenge (Chang et al., 2007; Singer et al., 2007). Further, the hepatic glycogen may have provided carbohydrates for glycolysis to maintain branchial ionic homeostasis in tilapia, gilthead sea bream, and rainbow trout in time-course experiments (Soengas et al., 1993; Sangiao-Alvarellos et al., 2005; Chang et al., 2007). The milkfish is a marine euryhaline species with branchial Na^+^/K^+^-ATPase (NKA), the major energy-requiring pump for maintaining ionic homeostasis, more highly expressed in FW-acclimated individuals than in SW-acclimated ones. Therefore, the energy demand for ionoregulation was higher in milkfish under hypo-osmotic stress (Jana et al., 2006; Tang et al., 2010; Kang et al., 2015) reported that milkfish fry acclimated to 25‰ seawater had the lowest level of hepatic glycogen, but exhibited the best growth performance (1.2% g day^−1^) and muscle protein content. It is obvious that changes in environmental salinity influence strategies of energy accumulation and growth performance in milkfish (Jana et al., 2006). The present study further revealed that the energy storage or utilization strategies of milkfish obviously vary depending on the environmental salinities to which they are acclimated. On the other hand, the acute utilization strategies of hepatic glycogen in milkfish for the entire body upon hypo-osmotic challenge are still unknown. Future studies will focus on the mechanism by which hepatic glycogen in milkfish is used for maintaining ionic homeostasis upon hypo-osmotic challenge.

### Temperature effects on glycogen utilization in the livers of milkfish

Low temperature appears to be an important factor to regulate glycogen storage in the crucian carp (*Carassius carassius*), a temperate species. A two-step cooling down procedure (from 18°C to 7.5°C maintained for 30 days, then from 7.5 to 2°C maintained until 60 days) induced hepatic glycogen storage, whereas the direct cooling-down (to 2°C) procedure did not change the glycogen content in the livers of the crucian carp (Varis et al., 2016). In the gilthead sea bream, a subtropical species, the reduction of feed intake was significantly correlated with environmental temperatures and liver glycogen deposition was observed in the low-temperature (18°C) group rather than in the normal-temperature (25°C) group (Couto et al., 2008; Enes et al., 2008). Similarly, reduction of feed intake was also observed in the FW- and SW-acclimated milkfish under hypothermal (18°C) adaptation. When environmental temperature decreased to 8°C, however, the energy utilization strategy of sea bream changed to the production of nonpolar and polar lipids, whereas the glycogen content in the livers was not significantly changed. In environments with much lower temperatures, the production of lipids was altered to maintain membrane fluidity, and glycogen storage decreased in the liver of the sea bream (Ibarz et al., 2010a; Melis et al., 2017). Being a tropical and herbivorous species, the milkfish cannot survive in temperatures lower than 15°C and continue to use carbohydrates as their main energy resource (Chiu and Benitez, 1981; Benitez, 1983; Hu et al., 2015). On the other hand, when silver catfish (*Rhamdia quelen*), another tropical species, were transferred from 20°C to 15°C, their hepatic glycogen degraded (Lermen et al., 2004). In the tropical white shrimp (*Litopenaeus vannamei*), the catabolism of glycogen was detected under cold stress (Zhou et al., 2011). In this study, hepatic glycogen degradation was also detected in FW-acclimated milkfish, but not in SW-acclimated milkfish, under hypothermal adaptation (18°C). In aquatic organisms, hepatic glycogen storage or catabolism was affected by external temperature. When out of the optimal temperature range, reduction of feed intake and hepatic glycogen degradation were reported in aquatic organisms from tropical, subtropical, or temperate habitats. In addition, anti-oxidative responses under hypothermal stress were found in the livers of the zebrafish (*Danio rerio*), Nile tilapia (*O. niloticus*), and gilthead sea bream (Ibarz et al., 2010b; He et al., 2015; Wu et al., 2015). This antioxidant mechanism is energy consuming (Espinosa-Diez et al., 2015; Huang et al., 2015). The glycogen may be used in glycolysis to produce ATP or by the pentose phosphate pathway (Stanton, 2012). Then, the antioxidant system works depending on production of NADPH from the pentose phosphate pathway. Under hypothermal adaptation, FW milkfish encounter more oxidative stress in the liver than that by SW milkfish (Chang et al., 2016a,b, 2017). Therefore, the response of milkfish upon hypothermal and salinity stress may be correlated with the elevation of energy requirements.

### Regulation of phosphorylation state of GP between FW and SW milkfish under cold stress

The activity of GP is regulated by the phosphorylation cycle, including the unphosphorylated T (tense)-state and phosphorylated R (relax)-state. The phosphorylation-state affected GP activity might be regulated by hormonal stimulation in the acute phase (Andersen and Westergaard, 2002; Milligan, 2003; Clow et al., 2008). The level of cortisol and glucagon-like peptide (GLP) in teleosts were suggested to play a role in elevating GP activity (Mommsen et al., 1999; Hallgen et al., 2003; Milligan, 2003). Barton and Peter (1982) found that the plasma cortisol levels of rainbow trout increased within 30 min and were maintained up to 4 h after rapid temperature decrease (from 10–11°C to 1°C), whereas they were recovered to the normal level after 24 h. Arjona et al. (2009) reported that plasma cortisol levels were increased 20-fold upon hypo-osmotic challenge in the Senegalese sole. In addition, 12°C exposure for 1 h induced an increase in the plasma cortisol levels in tilapia, whereas 19°C cold exposure did not cause this change (Chen et al., 2002). In addition, the GLP increased blood glucose levels via activation of gluconeogenesis in the livers of teleosts (Mojsov, 2000). The GLP levels of carp (*Cyprinus carpio*) blood were upregulated under acute changes in temperatures (Navarro et al., 2002). In the present study, after 12 h cold-exposure, increasing protein abundance of GP and phosphorylation levels along with an increase in GPase activity and decrease in glycogen content were found in the livers of FW milkfish, but not in SW milkfish. The total GP (R+T state) and GP (R state) allowed for identification of the elevated phosphorylation state of GP upon hypothermal stress in FW milkfish; however, no such change was noted in SW milkfish. The FW-acclimated juvenile milkfish had lower cold tolerance than the SW-acclimated fish (Kang et al., 2015). According to Milligan (2003), stress hormones might induce hepatic glycogen degradation to meet the glucose demands in FW milkfish under cold stress. Moreover, the GP activity in SW milkfish livers was not significantly different under cold stress, whereas downregulation of hepatic *Ccpygl* immediately after cold exposure in SW milkfish might be regulated via insulin or an insulin-like hormone (Klover and Mooney, 2004; Polakof et al., 2012).

## Conclusion

The results of the molecular, biochemical, and enzymatic analyses in this study illustrated the hypothermal effects on salinity-dependent glycogen utilization strategies in juvenile milkfish. In hypothermal acclimation, the feed intake of milkfish reduced in both FW and SW milkfish. However, only the FW milkfish upregulated hepatic glycogen catabolism to supply the energy required for acclimation to hypothermal stress. The phosphorylation state, protein abundance, and transcript levels of GP in milkfish livers were further found to be adjustable in the acute phase upon hypothermal challenge, corresponding to the salinity of the acclimated environment.

## Author contributions

C-HC and T-HL conceived the study. C-HC, J-JH, and T-HL discussed and designed the experiments. C-HC, J-JH, C-YY, and L-YH carried out the animal culture. C-HC, J-JH, and C-YY performed the experiment. C-HC and T-HL contributed to writing the manuscript. Both authors read and approved the final manuscript. C-HT glycogen phosphorylase (GP) antibody and test of experimental condition of GP immunoblotting.

### Conflict of interest statement

The authors declare that the research was conducted in the absence of any commercial or financial relationships that could be construed as a potential conflict of interest.
