# Supplementary material for: Salinity Effects on Strategies of Glycogen Utilization in Livers of Euryhaline Milkfish (Chanos chanos) under Hypothermal Stress
Source: Front Physiol. 2018 Feb 12;9:81. doi: 10.3389/fphys.2018.00081 (PMC5816346; doi:10.3389/fphys.2018.00081)

**Supplementary Table 1 Reference proteins from NCBI database for glycogen phosphorylase protein family phylogenetic analysis**

| **Species** | **Protein name** | **Accession number** |
| --- | --- | --- |
| *Homo sapiens* | Muscle glycogen phosphorylase | AAC52081.1 |
|  | Liver glycogen phosphorylase | AAC17450.1 |
|  | Brain glycogen phosphorylase | AAA59597.1 |
|  | Glycogen phosphorylase, muscle form isoform 1  Glycogen phosphorylase, liver form isoform 1 | NP_005600.1  NP_002854.3 |
|  | Glycogen phosphorylase, brain form | NP_002853.2 |
|  | Liver glycogen phosphorylase | AAC23504.1 |
|  | Muscle glycogen phosphorylase | AAC17451.1 |
| *Rattus norvegicus* | Glycogen phosphorylase, brain form | NP_037320.1 |
|  | Glycogen phosphorylase, liver form | NP_071604.1 |
|  | Glycogen phosphorylase, muscle form | NP_036770.1 |
| *Mus musculus* | Glycogen phosphorylase, muscle form | NP_035354.1 |
|  | Glycogen phosphorylase, liver form | NP_573461.2 |
|  | Glycogen phosphorylase, brain form | NP_722476.1 |
|  | Muscle glycogen phosphorylase | EDL33244.1 |
|  | Brain glycogen phosphorylase | AAH35283.1 |
|  | Liver glycogen phosphorylase | AAH13636.1 |
| *Danio rerio* | Glycogen phosphorylase, brain form | NP_997974.2 |
|  | Glycogen phosphorylase, liver form | NP_001008538.1 |
|  | Glycogen phosphorylase, muscle form | NP_001018464.1 |
| *Scleropages formosus* | Glycogen phosphorylase, brain form | KPP67981.1 |
|  | Glycogen phosphorylase, liver form | KPP79726.1 |
|  | Glycogen phosphorylase, muscle form | KPP69578.1 |
| *Larimichthys crocea* | Glycogen phosphorylase, liver form | KKF13421.1 |
|  | Glycogen phosphorylase, muscle form | KKF09517.1 |
| *Ictalurus punctatus* | Glycogen phosphorylase, liver form | AHH39573.1 |
| *Salmo salar* | Glycogen phosphorylase, brain form | NP_001167051.1 |
|  | Glycogen phosphorylase, liver form | XP_014067148.1 |
|  | Glycogen phosphorylase, muscle form | ACN10567.1 |

**Fig. S1 Representative immunoblots of GP in liver of milkfish.**


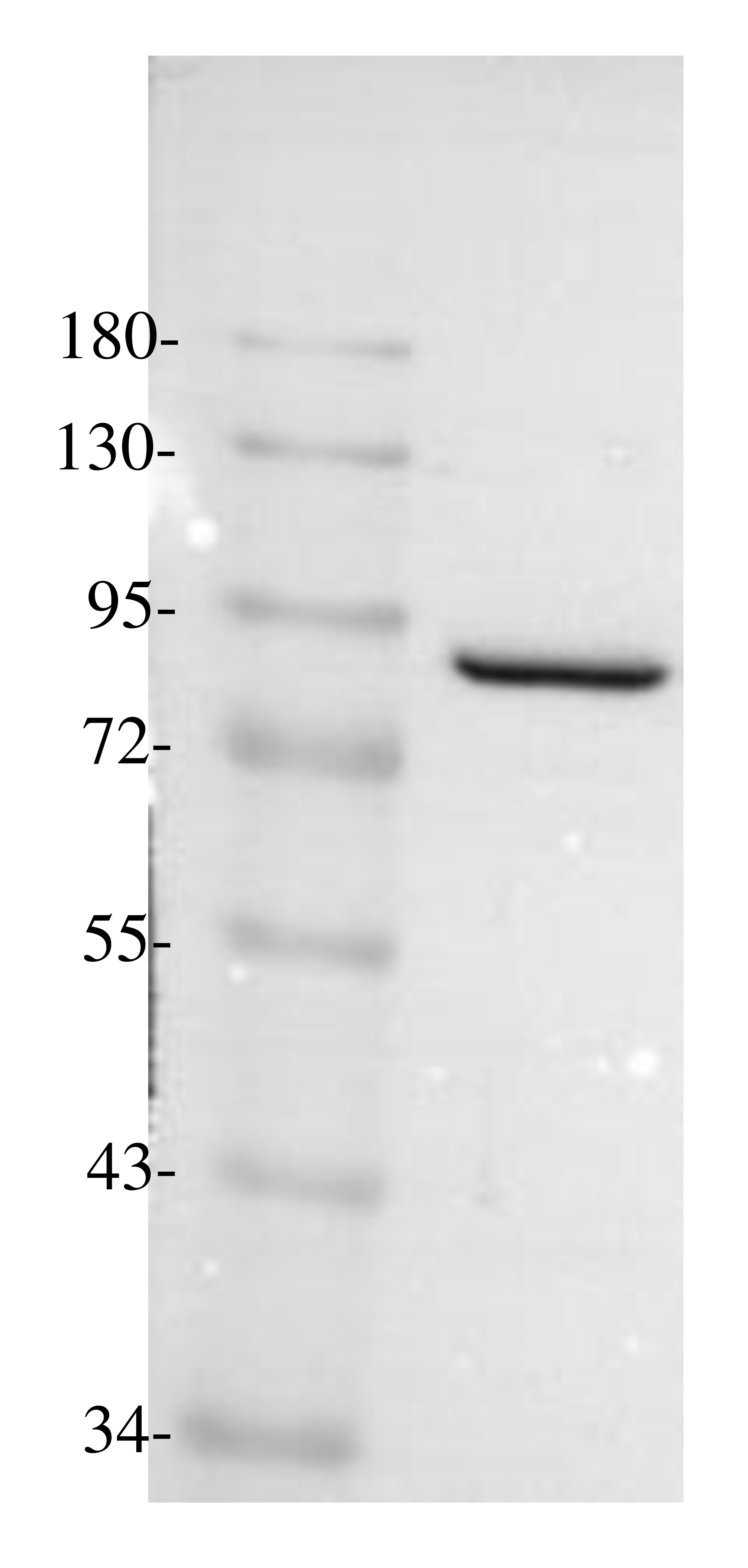


**Fig. S2 Phylogenetic analysis of Pygb, Pygm, and Pygl proteins based on amino acid sequences using the maximum likelihood method.** The results were confirmed by 1000 bootstraps. Sequence accession numbers were listed in the Supplementary Table 1.


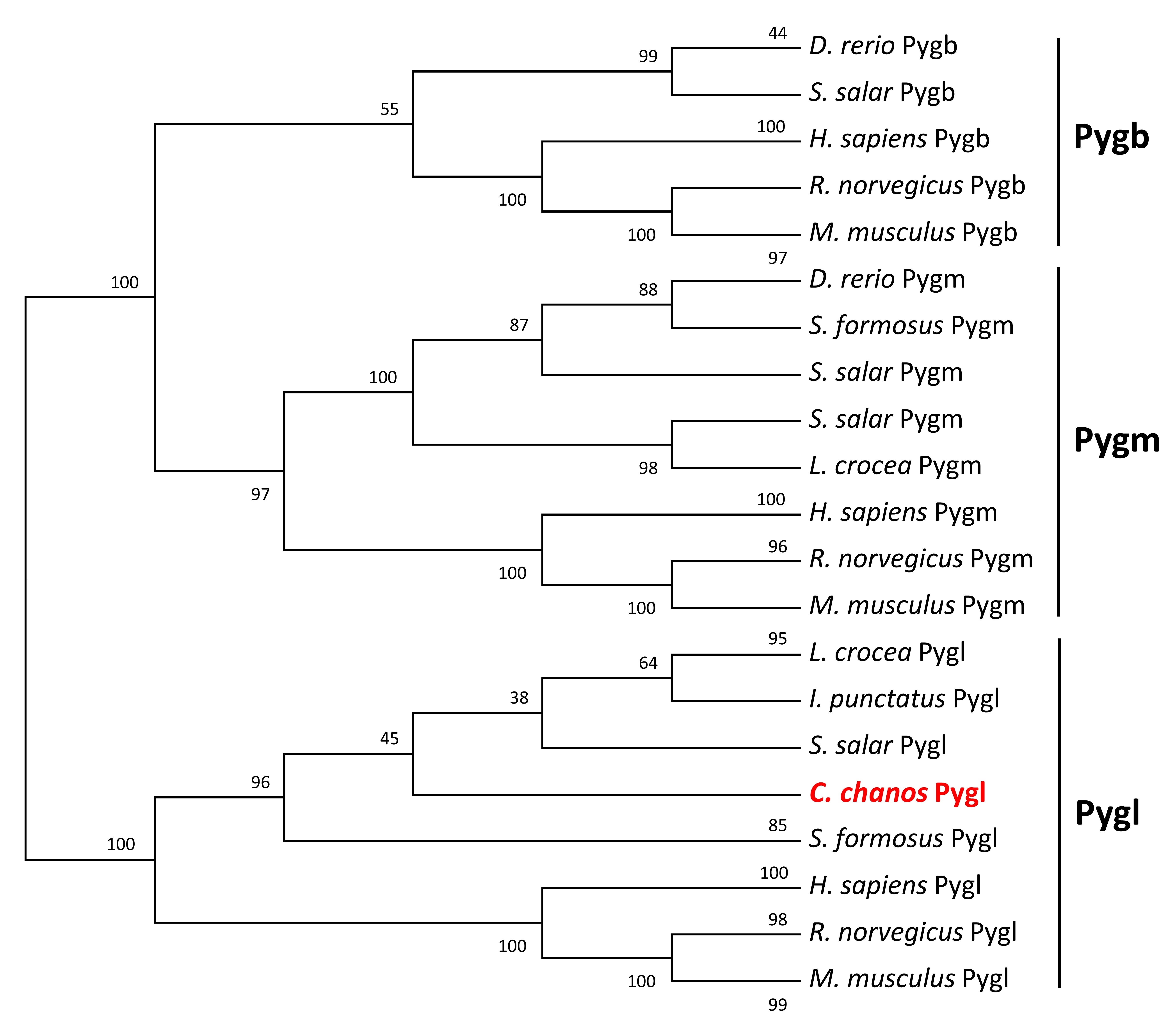

Supplement: Supplementary file 1 [file Table1.DOCX]
