# Supplementary figures and images for: Salinity Effects on Strategies of Glycogen Utilization in Livers of Euryhaline Milkfish (Chanos chanos) under Hypothermal Stress
Source: Front Physiol. 2018 Feb 12;9:81. doi: 10.3389/fphys.2018.00081 (PMC5816346; doi:10.3389/fphys.2018.00081)

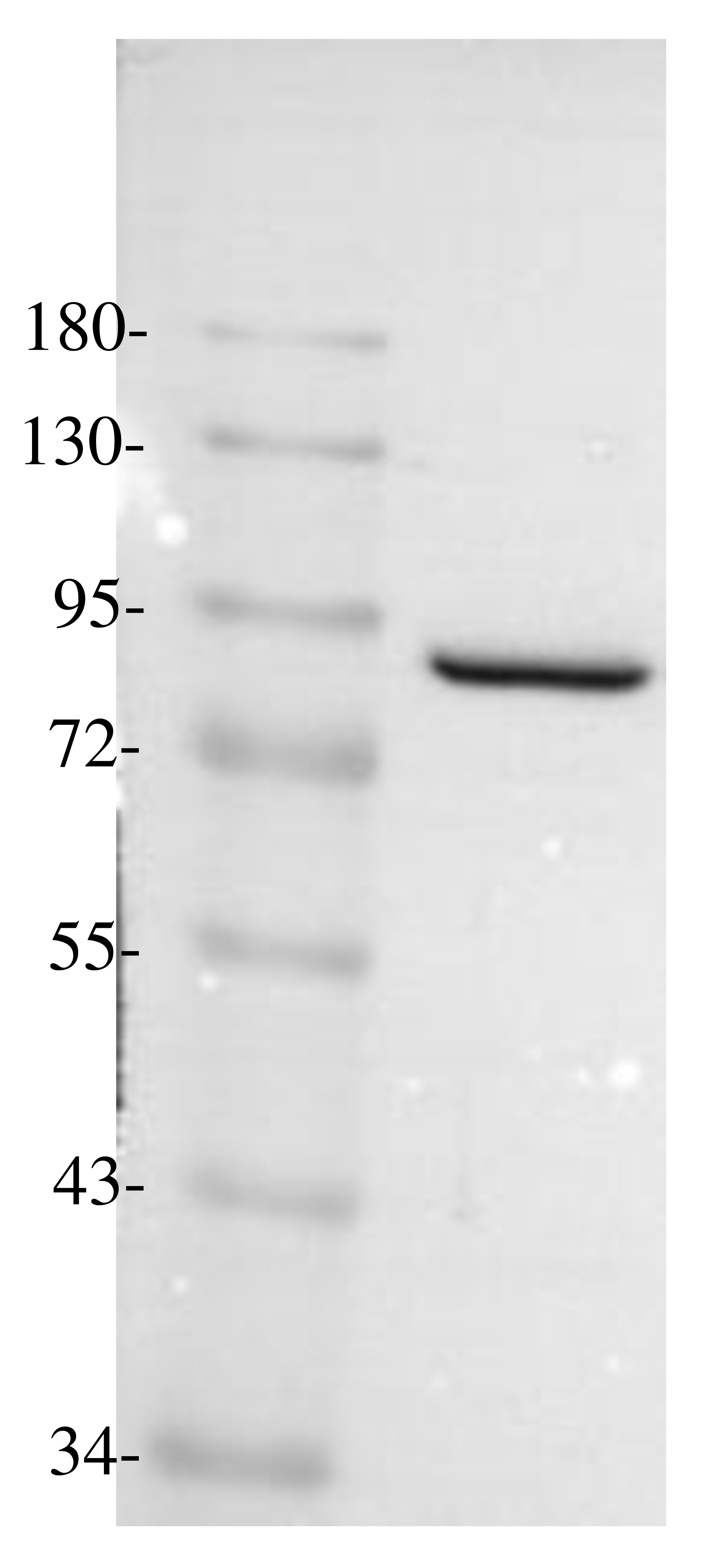

Supplement: Supplementary file 2 [file Image1.JPEG]

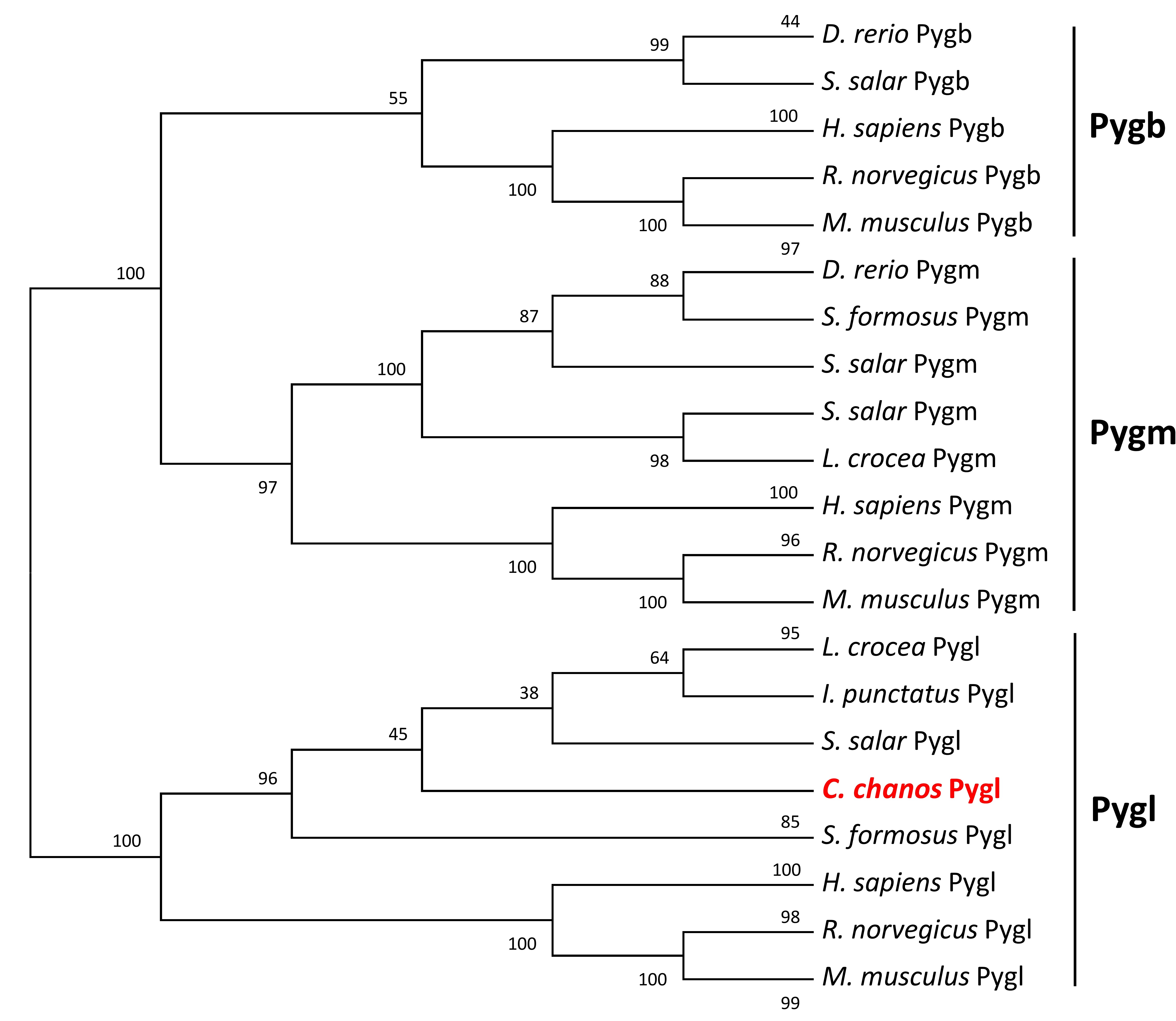

Supplement: Supplementary file 3 [file Image2.JPEG]
